# Melatonin modulates Nrf2 activity to protect porcine pre‐pubertal Sertoli cells from the abnormal H_2_O_2_ generation and reductive stress effects of cadmium

**DOI:** 10.1111/jpi.12806

**Published:** 2022-05-24

**Authors:** Desirée Bartolini, Iva Arato, Francesca Mancuso, Daniela Giustarini, Catia Bellucci, Carmine Vacca, Maria Chiara Aglietti, Anna Maria Stabile, Ranieri Rossi, Gabriele Cruciani, Mario Rende, Riccardo Calafiore, Giovanni Luca, Francesco Galli

**Affiliations:** ^1^ Department of Pharmaceutical Sciences University of Perugia Perugia Italy; ^2^ Department of Medicine and Surgery, Section of Human, Clinical and Forensic Anatomy University of Perugia Perugia Italy; ^3^ Department of Medicine and Surgery University of Perugia Perugia Italy; ^4^ Department of Biotechnology, Chemistry and Pharmacy University of Siena Siena Italy; ^5^ Department of Chemistry, Biology and Biotechnology University of Perugia Perugia Italy; ^6^ Division of Medical Andrology and Endocrinology of Reproduction Saint Mary Hospital Terni Italy; ^7^ Department of Medicine and Surgery, International Biotechnological Center for Endocrine, Metabolic and Embryo‐Reproductive Translational Research (CIRTEMER) University of Perugia Perugia Italy

**Keywords:** cadmium, glutathione, melatonin, NFkB, Nrf2, reductive stress, Sertoli cells

## Abstract

Melatonin (MLT) is a cytoprotective agent holding potential to prevent cadmium (Cd) toxicity and its impact in testicular function and fertility. In this study, we explored such potential in porcine pre‐pubertal Sertoli cells (SCs). Cd toxicity resulted in impaired SC viability and function, abnormal cellular H_2_O_2_ generation and efflux, and induction of reductive stress by the upregulation of Nrf2 expression and activity, cystine uptake and glutathione biosynthesis, glutathione‐S‐transferase P (GSTP) expression, and protein glutathionylation inhibition. Cd toxicity also stimulated the activity of cellular kinases (MAPK‐ERK1/2 and Akt) and NFkB transcription factor, and cJun expression was increased. MLT produced a potent cytoprotective effect when co‐administered with Cd to SCs; its efficacy and the molecular mechanism behind its cytoprotective function varied according to Cd concentrations. However, a significant restoration of cell viability and function, and of H_2_O_2_ levels, was observed both at 5 and 10 μM Cd. Mechanistically, these effects of MLT were associated with a significant reduction of the Cd‐induced activation of Nrf2 and GSTP expression at all Cd concentrations. CAT and MAPK‐ERK1/2 activity upregulation was associated with these effects at 5 μM Cd, whereas glutathione biosynthesis and efflux were involved at 10 μM Cd together with an increased expression of the cystine transporter xCT, of cJun and Akt and NFkB activity. MLT protects SCs from Cd toxicity reducing its H_2_O_2_ generation and reductive stress effects. A reduced activity of Nrf2 and the modulation of other molecular players of MLT signaling, provide a mechanistic rational for the cytoprotective effect of this molecule in SCs.

## 1 INTRODUCTION

Infertility affects around 15% of couples of childbearing age in industrialized countries.^1^ Since a male factor appears in about 30% of the cases, and in 20% both male and female factors are involved, cumulatively approximately 50% of cases of infertility involve the male partner.^2^ Unfortunately, the etiology of male infertility is still unknown in 30%–40% of cases.^3^

Environmental pollution is a possible risk factor of idiopathic male infertility. Heavy metals, such as lead, mercury, and cadmium (Cd), are pollutants representing an emerging health concern worldwide with proposed adverse effects on fertility.^4^, ^5^, ^6^, ^7^ Cd is a ubiquitous pollutant mainly deriving from industrial activities that cause severe toxicity in various organs, including the testis.^5^, ^8^, ^9^, ^10^ In fact, severe impairment of the male germ cells have been described both in rodents^11^ and humans exposed to Cd, often leading to azoospermia.^12^

Sertoli cells (SCs), representing the only somatic cells within the seminiferous tubules, can be considered the conductor of spermatogenesis and the guardian of germ cell integrity.^13^ The contribution of SCs to spermatogenesis is based on the production of critical factors (extracellular matrix components, growth factors and many proteins, such as transferrin, clusterin, and the stem cell factor), necessary for the successful development of spermatogonia up to the stage of the spermatozoon.^14^, ^15^ Furthermore, SCs contribute to the assemblage of the basal membrane that provides mechanical support to somatic as well as to undifferentiated germ cells, and governs several steps of the spermatogenesis.^16^ Finally, SCs can be considered the immunological sentinels of spermatogenesis. In fact, immune‐privileged SCs protect germ cells from the attack of immune cells, both sequestering these auto‐antigenic cells behind the physical BTB/SC barrier and secreting immunomodulatory molecules (such as IDO, TGFBeta, and clusterin).^17^ A previous work^5^ demonstrated that even low micromolar concentrations (5 μM) of Cd interfere with key functional parameters of superior mammalian SCs, such AMH, and inhibin B secretion, that can be considered sensitive biomarkers of the in vitro toxicity of this metal in these cells.

In light of the above, is apparent the need to provide new therapies for male infertility due to heavy metal exposure.

Melatonin (MLT), a hormonal substance exhibiting strong antioxidative and antiapoptotic properties,^18^, ^19^, ^20^ has been proved to hold great potential as cytoprotective agent in a range of tissues and cell models exposed to Cd toxicity, including the rat bone, ovaries, human MSC,^21^, ^22^, ^23^ and even mammalian testes.^24^, ^25^, ^26^, ^27^ However, cellular and mechanistic aspects of this effect of MLT in testes remain poorly understood. Considering the aforementioned important role of SC in testicular integrity and function, in this study we investigate for the first time the cytoprotective effect of MLT against Cd toxicity in porcine pre‐pubertal SCs. These cells show many similarities to human SCs, holding great potential as experimental model to study alterations of spermatogenesis and male fertility that occur early in the lifespan, as well as to develop xenotransplantation and cell therapy protocols.^28^, ^29^, ^30^, ^31^, ^32^

Because both the toxicity of Cd and the cytoprotective function of MLT involve redox‐dependent mechanisms,^20^, ^33^ the activity of Nuclear factor‐erythroid factor 2‐related factor 2 (Nrf2) transcription factor, a key player of the cellular stress response to electrophiles,^34^, ^35^ was explored for the first time in these cells together with other redox‐sensitive transcription factors important in MLT signaling, such as NFkB,^36^ and some redox homeostasis and detoxification genes downstream of these transcriptional hubs, including catalase (CAT) and the glutathione system.^37^

## 2 MATERIALS AND METHODS

### 2.1 Isolation and characterization of SCs

Animal studies were conducted in agreement with the guidelines adopted by the Italian Approved Animal Welfare Assurance (A‐3143‐01) and European Communities Council Directive of November 24, 1986 (86/609/EEC). The experimental protocols were approved by the University of Perugia. Number 3 Danish Duroc pre‐pubertal pigs (15–20 days old) underwent bilateral orchidectomy after general anesthesia with ketamine (Ketavet 100; Intervet), at a dose of 40 mg/kg, and dexmedetomidine (Dexdomitor, Orion Corporation, Finland), at a dose of 40 g/kg, and were used as SCs donors. Specifically, pure porcine pre‐pubertal SCs were isolated, and characterized according to previously established methods.^38^

### 2.2 SCs culture and treatments

SCs were maintained at 37°C in a 5% CO_2_ humidified atmosphere in the absence (unexposed ‐control group) or presence of 5 or 10 µM CdCl_2_ (Sigma Chemical Co.) and/or 50 nM MLT (Sigma‐Aldrich) for 48 h in HAMF12 (Euroclone) supplemented with 0.166 nM retinoic acid (Sigma‐Aldrich Co.) and 5/500 ml of Insulin‐Transferrin‐Selenium (ITS) + Premix (Cat. No. 354352; Corning).

### 2.3 Cell viability

Cell viability was determined in SCs (1 × 10^5^ cells/well) seeded in 96‐well plates and exposed to CdCl_2_ and/or MLT as described earlier, using the probe of mitochondrial dehydrogenase activity 3‐(4,5‐dimethylthiazol‐2‐yl)‐2,5‐diphenyltetrazolium bromide (MTT test).^32^ Data were reported as mean ± SD of eight replicates obtained from four independent experiments.

### 2.4 Reverse transcriptase‐polymerase chain reaction analysis

AMH and inhibin B determinations were performed by reverse transcriptase‐polymerase chain reaction (RT‐PCR) as previously described in Arato et al.^39^ employing the primers listed in Table 1. Total RNA was extracted using the TRIzol reagent (Sigma‐ Aldrich), and quantified by reading the optical density at 260 nm. In detail, 2.5 μg of total RNA was subjected to reverse transcription (RT, Thermo Scientific) to a final volume of 20 μl. We performed the qPCR with the use of 25 ng of the cDNA obtained by RT and an SYBR Green Master Mix (Stratagene). This procedure was performed in an Mx3000P cycler (Stratagene), using FAM for detection and ROX as the reference dye. We normalized the mRNA level of each sample against β‐actin mRNA and expressed it as fold changes versus the levels in the control group.

**Table 1:** Primer sequences for PCR analyses.

Gene	Forward sequences (5′–3′)	Reverse sequences (5′–3′)
AMH	GCGAACTTAGCGTGGACCTG	CTTGGCAGTTGTTGGCTTGATATG
Inhibin B	CCGTGTGGAAGGATGAGG	TGGCTGGAGTGACTGGAT
β‐actin	ATGGTGGGTATGGGTCAGAA	CTTCTCCATGTCGTCCCAGT

### 2.5 AMH and inhibin B secretion assays

Aliquots of culture media from unexposed and Cd‐ and/or MLT‐exposed SCs were collected and stored at −20°C for AMH determination by AMH Gen II enzyme‐linked immunosorbent assay (ELISA), Beckman Coulter (intra‐assay CV = 3.89%; inter‐assay CV = 5.77%) and inhibin B (Inhibin B Gen II ELISA, Beckman Coulter; intra‐assay CV = 2.81%; inter‐assay CV = 4.33%) secretion levels as previously described.^40^ ELISA, Beckman Coulter; intra‐assay CV = 3.89%; inter‐assay CV = 5.77%) and inhibin B (Inhibin B Gen II ELISA, Beckman Coulter; intraassay CV = 2.81%; inter‐assay CV = 4.33%) secretion levels as previously described.^40^

### 2.6 Intracellular and extracellular reactive oxygen species and catalase activity

Cellular reactive oxygen species (ROS) was assessed using the fluorescent probe 2′,7′‐ dichlorofluorescein diacetate (DCFH‐DA; Sigma‐Aldrich).^41^ The specificity of the assay for the detection of H_2_O_2_ and the effect of thiol depletion on cellular stress parameters were verified as described in Bartolini et al.^42^ uploading the cells with PEG‐catalase (PEG‐CAT; 50 U/ml, Sigma‐Aldrich), a membrane‐permeable form of this enzyme.

Extracellular H_2_O_2 _was determined with a microplate assay procedure utilizing the Amplex™ Red Hydrogen Peroxide/Peroxidase Assay Kit (Invitrogen).^43^ Briefly, 100 µl cell supernatant was placed in 96‐well plates and incubated in a humidified atmosphere with 5% CO_2_ at 37°C, with HRP (1 U/ml) and Amplex Red for 10 min. Then, the fluorescence was measured using a DTX880 Multimode Detector microplate reader (Beckman Coulter). The assay was calibrated with authentic H_2_O_2_ and two quality control samples (QCs). Data were mean ± SD of four independent experiments run in eight replicates.

CAT activity was measured in 0.5 mg of total proteins of cell lysates by ammonium molybdate titration of the enzymatic decomposition of H_2_O_2_ reaction substrate using a colorimetric assay kit (Elabscience Biotechnology Inc.). The yellowish complex formed by the ammonium molybdate reaction with the peroxide was measured at 405 nm.

### 2.7 Immunofluorescence and quantitative confocal microscopy analysis

After treatment, SCs in 96 well plates flat‐bottomed black polystyrene wells (with micro‐clear bottom, Corning) were fixed with 10% neutral formalin (Leica), and then washed three times with PBS (Lonza) and permeabilized with Triton X‐100 0,5% in PBS for 5 min at room temperature (RT). Blocking was carried out with 3% FBS for 20 min at RT under slow stirring. The plates were washed with distilled water (Euroclone) and then the incubation with the primary antibody was carried out in 3% FBS for 1 h at RT and under slow stirring. After three cycles of washing in distilled water, the labeled secondary antibodies (1:1000 in 3% FBS) and the fluorescent probe utilized to stain the nuclei DAPI (Life Technologies) (1:3000) were added in 3% FBS and incubated for 1 h in the dark. The plates were rinsed with four washes in distilled water and AlexaFluor 488 (Life Technologies) was added (1:1000 in PBS) and the plates were incubated for 40 min in the dark under stirring to stain actin filaments. Finally, after six washes in sterile distilled water and the plates were ready for quantitative fluorescence analysis (from 63 to 72 fields/well) and image acquisition by Operetta CLS system (Perkin Elmer) equipped with a 40X water immersion objective. The primary antibodies used in this study were: Nrf2 Polyclonal Antibody (Cell Signaling, 1:50); GSTP1 rabbit polyclonal antibody (HPA019869, Atlas Antibodies, 0.5 µg/ml). The secondary antibody was Texas Red (TR) anti‐rabbit IgG (1:2000, Invitrogen).

### 2.8 Immunoblot

SCs were prepared for immunoblot analysis as described in Bartolini et al.^44^ Briefly, proteins were quantified in the cellular extracts with bicinchoninic acid (BCA) assay and then 20 μg of proteins were loaded onto 10% SDS–PAGE minigels (Novex WedgeWell Tris‐Glycine gel, Invitrogen). After separation, these proteins were blotted and immobilized on a nitrocellulose membrane that was incubated with 5% skim milk in Tris‐buffered saline (TBS; 20 mM Tris base, 150 mM NaCl, pH 7.4) and 0.1% Tween‐20 for 2 h at room temperature. The blots were incubated with primary antibodies at 4°C overnight, with constant shaking and then washed twice with TBS. The primary antibodies were: anti‐xCT (ab175186; 1:1000) from Abcam, anti‐β‐actin (#3700; 1:1000) and anti‐GCLC (E‐AB‐52359, 1:2000) from Elabscience, and anti‐β‐actin (#3700; 1:1000), anti‐Phospho‐NFkB (#4806; 1:1000), anti‐NFkB (#8242; 1:1000), anti‐*c*‐Jun (#9165; 1:1000), anti‐Phosho‐ERK1/2 (#4370; 1:1000), anti‐ERK1/2 (#4695; 1:1000), Phospho‐AKT (#4060; 1:1000), anti‐AKT (#9272; 1:1000), anti‐GAPDH (#2118; 1:2000) and anti‐α/β‐Tubulin (#2148; 1:1000) from Cell Signaling Technology. The secondary antibodies were anti‐rabbit (#7074) or anti‐mouse (#7076) IgG (1:2000) horseradish peroxidase‐linked (Cell Signaling Technology). Protein bands were detected using an ECL Clarity or ECL Clarity Max (BioRad). Quantification of bands was performed with a Gel‐Pro Analyzer; protein expression levels were normalized against housekeeping proteins.

### 2.9 Protein glutathionylation (PSSG)

Semiquantitative PSSG determination was carried out by immunoblot as reported in Bartolini et al.^45^ Briefly, after Cys residues alkylation with NEM, cellular proteins (20 μg) were fractionated by SDS–PAGE under nonreducing conditions using 4%–12% pre‐casted gels (Invitrogen) and then transferred to a nitrocellulose membrane (Thermo Fisher Scientific) for immunoblot analysis. An anti‐GSH primary antibody (1:1000, Abcam) and an anti‐mouse IgG HRP‐linked secondary antibody (1:2000, Cell Signaling Technology) were utilized for PSSG identification.

### 2.10 Thiols and disulfide analysis

Cellular thiols were assessed by HPLC analysis with fluorescence detection after derivatization with monobromobimane (mBrB, Calbiochem) as reported in Bartolini et al.^45^ and references therein. For disulfide analysis, aliquots of samples were reacted with N‐ethylmaleimide (Sigma‐Aldrich) to mask reduced thiols and then with dithiothreitol (DTT, Sigma‐Aldrich) to reduce intramolecular disulfide bridges.

### 2.11 Statistical analysis

Data distribution and differences between mean data were assessed by one‐way analysis of variance (ANOVA) followed by Tukey's test for multiple comparison or Student's *t*‐test for paired or unpaired data. Statistical analysis was performed using GraphPad Prism (v.6.0).

## 3 RESULTS

### 3.1 Effect of Cd and MLT on porcine SCs viability and ROS production

**Figure 1:**
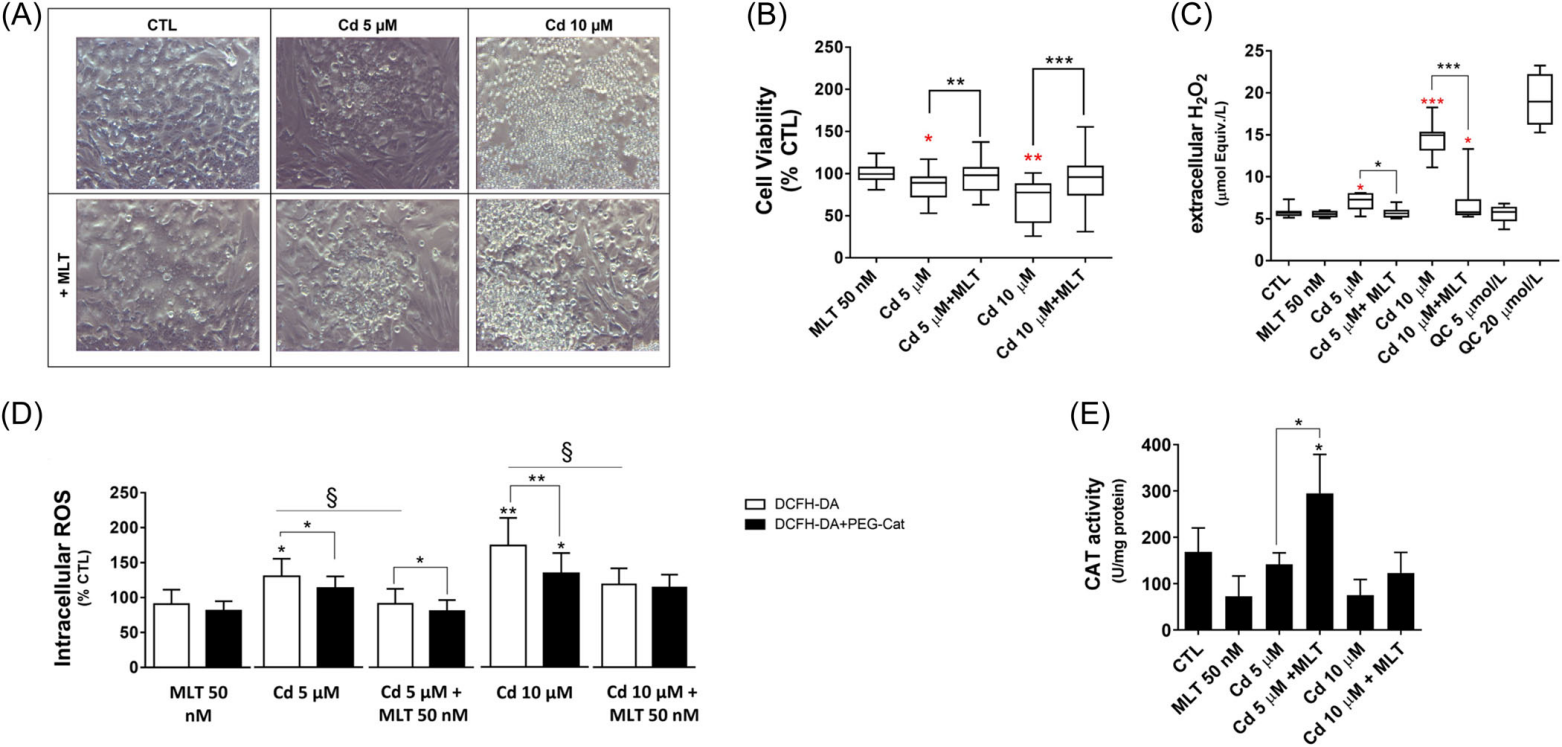
Effect of cadmium (Cd) and melatonin (MLT) on Sertoli cells (SCs) viability, ROS production, and catalase activity. SCs were treated with 5 or 10 µM Cd and/or with 50 nM MLT for 48 h as described in the section Methods. (A) SCs morphology was assessed by optical microscopy (magnification 40X), and (B) cell viability was assessed by MTT test; data in the different treatments were expressed as percentage of viable cells relative to control (untreated) cells (% CTL). *t*‐test: *p < .05; **p < .001 versus CTL (red); Cd versus Cd + MLT; *p < .05; **p < .001; ***p < .0001; one‐way ANOVA: Cd 10 µM versus Cd 10 µM + MLT 50 nM ***p < .0001. (C) Cellular ROS efflux was measured with Amplex Red fluorescent probe that is selective for H_2_O_2_. One‐way ANOVA: *p < .05; ***p < .0001 versus CTL (red); *p < .05; ***p < .0001 versus Cd + MLT. (D) Intracellular ROS were assessed with DCFH‐DA fluorescent probe and PEG‐catalase was utilized to identify the quota of DCFH‐DA oxidation associated with H_2_O_2_ production. One‐way ANOVA: *p < .05; **p < .001 versus CTL; ^§^ *p* < .05. QC (quality control experiments). (E) Catalase enzyme activity was assessed in cell lysates (0.5 mg of total proteins) using a spectrophotometric assay procedure and the results were expressed as U/mg of protein. *t*‐test: *p < .05; CTL versus treatments and Cd versus Cd + MLT.

According to previous work by some of us,^5^ Cd showed a significant and concentration‐dependent toxicity in SCs, reducing cell viability (Figure 1A,B), and stimulating ROS production (Figure 1C,D). PEG‐CAT incorporation into SCs demonstrated that such ROS include H_2_O_2_ and other species (Figure 1D). Worth of note is that these cells promptly release in the extracellular medium the load of H_2_O_2_ generated by the effect of Cd (Figure 1C). Apparently, this efflux mechanism maintains the intracellular levels of this species relatively stable, even if increased (Figure 1D), during the response to Cd toxicity. These findings highlight the phagocyte‐like properties of these cells upon stimulation of stress pathways by the exposure to either xenobiotics and oxidative stress,^27^, ^46^, ^47^, ^48^ as well as to physiological stimuli, such as during ectoplasmic specialization to support all phases of germ cell development and maturity.^49^, ^50^

MLT co‐treatment significantly prevented the effects of Cd on cell viability and abnormal ROS production (Figure 1C,D), confirming the cytoprotective function and antioxidant properties of this hormonal substance^20^ and its relevance in preventing Cd toxicity.^21^ The reduction of H_2_O_2_ efflux was very important (Figure 1C) and combined with the effect on the cellular levels of this species (Figure 1D), an induction effect of MLT on cellular H_2_O_2_‐scavenging systems can be hypothesized.

To explore this mechanistic interpretation, the enzymatic activity of CAT, the main H_2_O_2_‐metabolizing system of the cell with proposed role in MLT antioxidant function,^36^, ^51^ was investigated. An increased CAT activity was demonstrated upon MLT treatment that was significantly in SCs exposed to 5 μM Cd (Figure 1E).

### 3.2 SCs functionality

AMH and inhibin B were investigated as biomarkers of SCs function^5^ in the Cd cytotoxicity model of this study.

**Figure 2:**
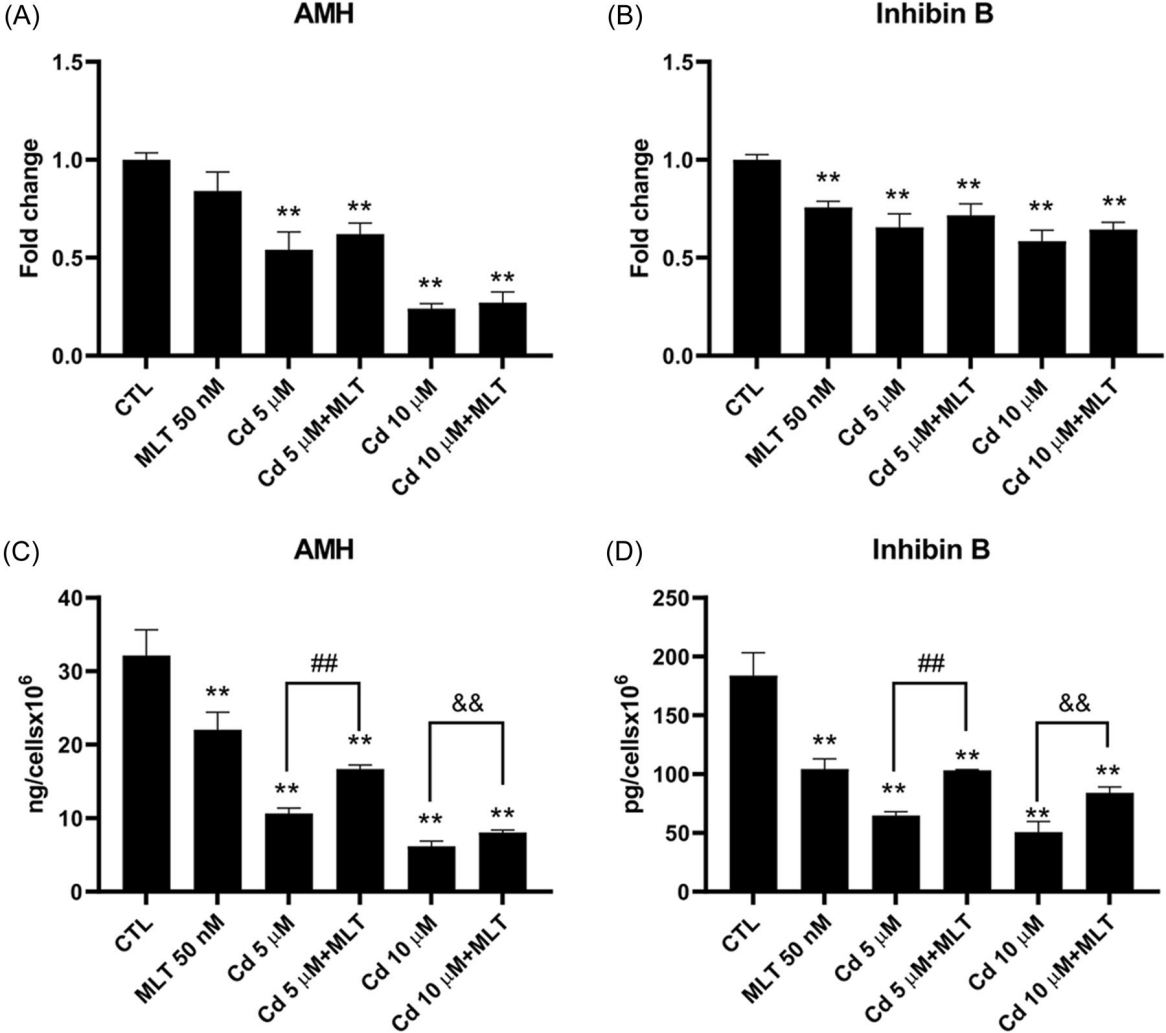
Effect of cadmium (Cd) and melatonin (MLT) on the Sertoli cells (SCs) functionality parameters AMH and inhibin B. Gene expression (upper panels) and secretion (bottom panels) of AMH (A, C) and inhibin B (B, D) were assessed by real‐time PCR and ELISA analysis, respectively, in SCs treated with CdCl_2_ (Cd) 5 or 10 µM and/or 50 nM MLT for 48 h. Data were as mean ± S.E.M. *t*‐test: **p < .001 versus unexposed SCs; ^##^ *p* < .001 versus Cd 5 µM and ^&&^ *p* < .001 versus Cd 10 µM of three independent experiments, each performed in triplicate.

Cd treatment significantly reduced the gene expression (Figure 2A,B) and secretion levels (Figure 2C,D) of these markers in SCs.

The co‐treatment with MLT compared to Cd treatment, showed a trend toward a recovery and a significant recovery of AMH and inhibin B gene expression and secretion levels, respectively, demonstrating the cytoprotective effect of MLT in SCs exposed to Cd toxicity at the functional level.

### 3.3 Nrf2, other transcription factors, MAPK‐ERK, and glutathione parameters

**Figure 3:**
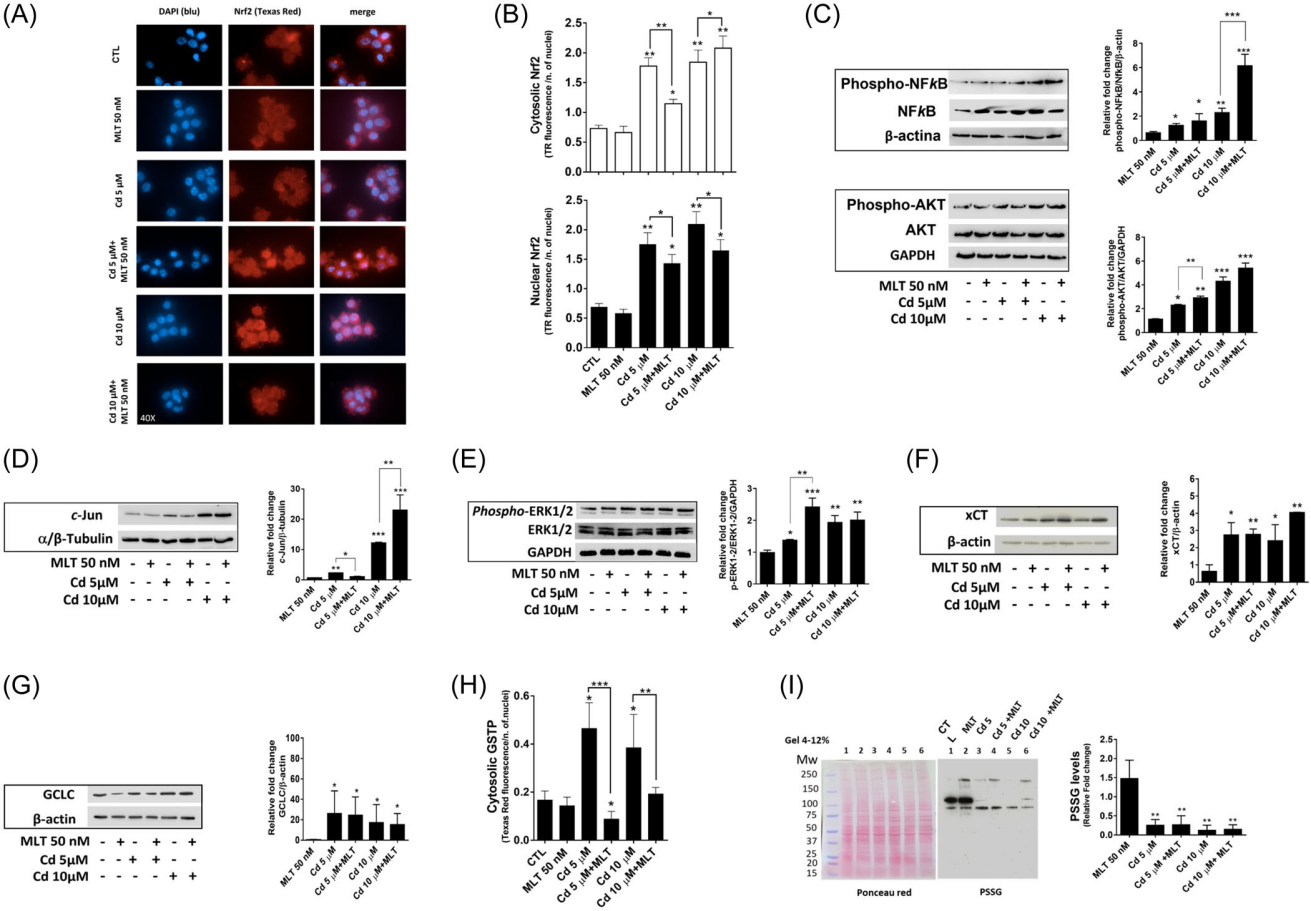
Effect of cadmium (Cd) and/or melatonin (MLT) on Nrf2, other transcription factors and signaling proteins, and glutathione‐related genes of Sertoli cells (SCs). (A) Confocal microscopy immunofluorescence analysis of Nrf2 nuclear translocation. Labeling of Nrf2 and nuclei was carried out with Texas Red (TR, red) and DAPI (blue) fluorophores, respectively. Magnification = 40X. (B) Cytosolic and nuclear levels of Nrf2 were determined by semi‐quantitative microplate confocal microscopy analysis. Immunoblot was used to assess (C) NFkB and AKT (D) *c*‐Jun (E) ERK1/2, (F) the membrane transporter involved in cystine uptake xCT, and (G) the catalytic subunit of glutathione biosynthesis enzyme gamma‐glutamylcysteine liase (GCLC). Densitometric analysis data were shown as relative expression of housekeeping proteins and protein expression of control tests in each series of experiments. (H) Cytosolic GSTP protein expression was determined by confocal microscopy as described in (A) and (B). (I) protein S‐glutathionylation (PSSG) was investigated by immunoblot; PSSG levels were normalized for the total cellular proteins and were expressed as fold change with respect to control cell mean levels. Data were as mean ± SD of three independent experiments. *p < .05; **p < .001; ***p < .0001.

Cd toxicity in SCs was characterized by the modulation of transcription factors with important role in the adaptive stress response, including a significant activation of Nrf2 expression and nuclear translocation (Figure 3A,B), NFkB protein phosphorylation (Figure 3C), and cJun expression (Figure 3D). Phosphorylation of AKT and MAPK‐ERK1/2 cellular kinases was also induced by Cd treatment (Figure 3C, lower panel, and Figure 3E, respectively).

Particularly, the activity of the transcription factors investigated in this study is important for the cell to adapt to redox stressors by the induction of antioxidant and detoxification genes, such as those of the glutathione defense system.^34^, ^52^ In fact, the effect of Cd on transcription factor activation was associated with the induction of xCT, GCLC, and GSTP protein expression (Figure 3F–H), whereas PSSG levels decreased (Figure 3I).

**Figure 4:**
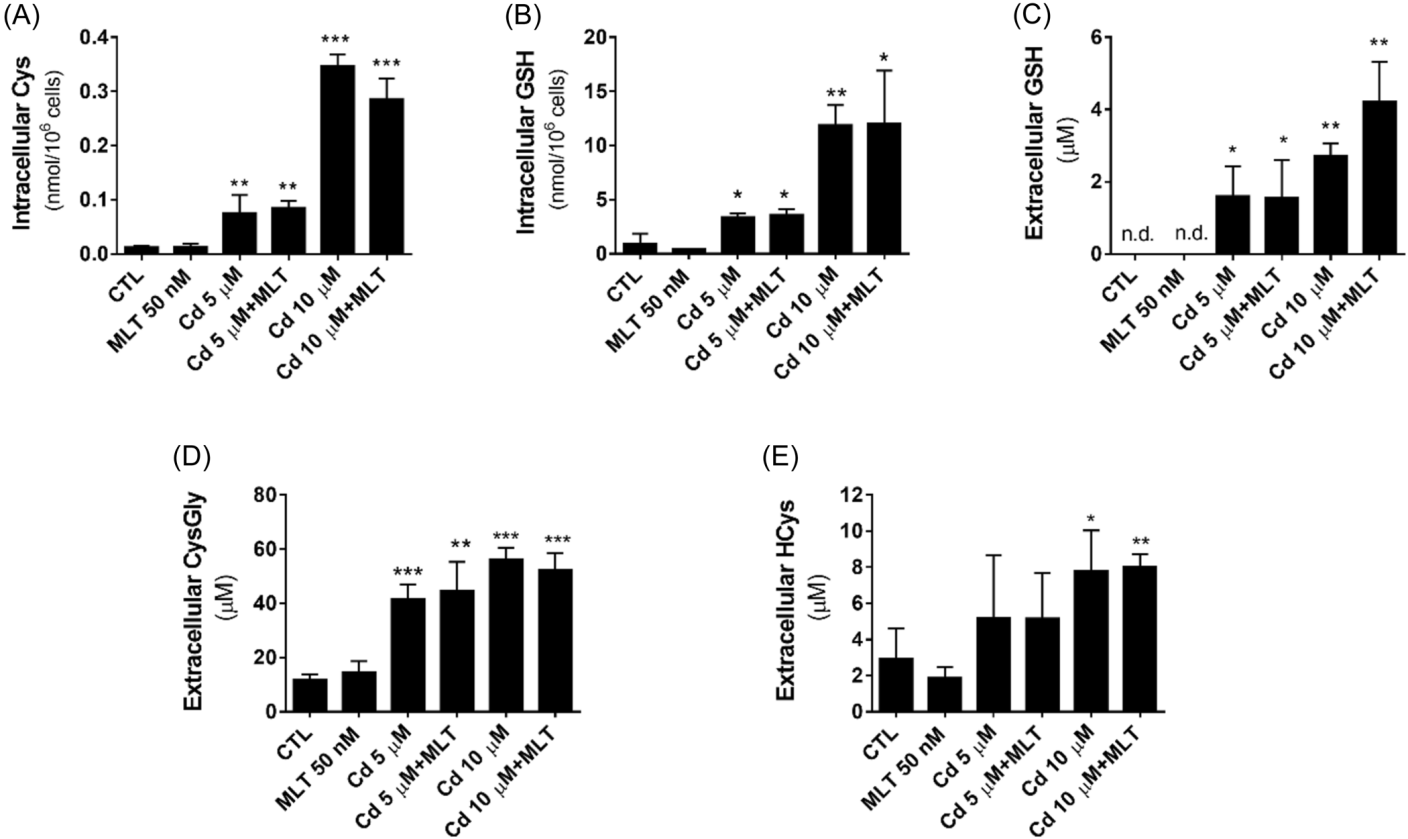
Levels of thiols in Sertoli cells (SCs) treated with CdCl_2_ (Cd) 5 or 10 µM and/or melatonin (MLT) 50 nM for 48 h. Intracellular (A, B) and extracellular (C, D, E) levels of GSH and other thiols measured by HPLC in SCs treated with Cd 5 or 10 µM and/or MLT 50 nM for 48 h. *t*‐test: *p < .01; **p < .005; ***p < .0001 versus Ctr.

The Cd‐induced stimulation of xCT and GCLC expression was consistent with a dose‐dependent modification of cellular thiols, ultimately resulting in a marked induction of the cellular uptake of Cys (Figure 4A) and its consequent utilization in the biosynthesis of GSH, the levels of which increased at the cellular level to be readily released in the extracellular milieu (Figure 4B,C, respectively). This stimulation effect of GSH metabolism also included an increased efflux of the GSH catabolism product CysGly and Hcys (Figure 4D,E, respectively).

Altogether these findings demonstrate the presence of a reductive stress response in SCs to the pro‐oxidant effect of Cd.

MLT treatment, when considered alone, did not affect Nrf2 and the other parameters associated with GSH metabolism, except than PSSG levels that increased compared to untreated control cells (Figure 3F). Whereas in Cd‐treated cells, MLT significantly reduced the expression and nuclear translocation of Nrf2 protein (Figure 3A,B), and decreased GSTP induction (Figure 3E). However, the other glutathione‐associated proteins were not affected, suggesting a specific role for the Nrf2‐GSTP axis in the cytoprotective response to MLT of Cd‐treated SCs.

Furthermore, xCT expression was significantly upregulated by MLT in SCs treated with 10 μM Cd (Figure 3C) that was associated with significantly higher biosynthesis and efflux of GSH (Figure 4B,C), by a higher utilization of cellular Cys (Figure 4A).

NFkB phosphorylation was markedly induced by MLT + Cd compared to Cd treatment (Figure 1C, upper panel), and the same was observed for Akt phosphorylation (Figure 3C, lower panel). Again, in comparison with Cd treatment, MLT reduced cJun protein expression of SCs treated with the lower dose of Cd used in this study (5 μM) whereas an increased expression was observed at the higher dose, that is, 10 μM (Figure 3D). MLT significantly increased MAPK‐ERK1/2 phosphorylation of cells treated with Cd 5 μM, but not with Cd 10 μM (Figure 3E).

## 4 DISCUSSION

MLT is a pleiotropic molecule involved in the physiological regulation of cellular processes and a potent cytoprotective agent with proposed application in several toxicities,^22^, ^53^ including that of heavy metals as Cd.^21^ Recent studies suggested that this hormonal substance may prevent Cd toxicity also in the mammalian testes.^24^, ^25^, ^26^ Such specific toxicity is associated with indices of oxidative stress and DNA damage in sperm, and currently it represents an important health concern and an emerging risk factor for human male infertility.^54^, ^55^

In an effort to develop effective chemopreventive treatments against Cd toxicity, recent studies by Li et al.^25^ and Venditti et al.^27^ described the role of MLT in protecting the integrity of BTB in rat testes exposed to this metalloid, with proposed effects on autophagy pathway activation and function of SCs.^27^

Considering these findings and the overall role of SCs as metronome of testicular homeostasis and spermatogenesis process, we herein investigated the effects of MLT in protecting SCs against Cd toxicity, with a focus on the redox‐dependent mechanism of action of this hormonal substance.

First, the MLT cytoprotective effect in SCs exposed to Cd toxicity was confirmed using functional biomarkers of these cells, such as the gene expression and secretion of AMH and inhibin B (Figure 2). AMH is exclusively secreted by SC, thus representing a useful markers of testis functionality during the pre‐pubertal period;^56^ inhibin B is a marker used in clinical practice to evaluate the presence and function of SCs during childhood.^57^ We observed a significant increase of AMH and inhibin B secretion upon treatment with MLT respect to the Cd‐exposed SCs, demonstrating a recovery effect on SCs function by this hormonal agent.

Therefore, the redox‐modulating function of MLT was investigated. MLT has been proposed to produce both direct and indirect (gene expression‐mediated) antioxidant effects, which could play a key role in preventing the pro‐oxidant effects of Cd and their damaging effect on testicular cells.^33^, ^54^, ^55^ Our findings in porcine pre‐pubertal SCs confirm that Cd toxicity is associated with the induction of a pro‐oxidant environment by sustained production and release in the extracellular space of H_2_O_2_ (Figure 1). This type of response highlights the phagocyte‐like phenotype of these cells and their capability to produce an oxidative burst to protect the testes from exogenous treats.

However, H_2_O_2_ is not only a cytotoxic product generated during phagocytic cell activation.^58^ It plays an important role as cellular messenger involved in the modulation of redox‐sensitive signal transduction and transcriptional proteins, including Nrf2, a key player of the cellular stress response to electrophiles.^34^, ^35^ According to this role of cellular H_2_O_2_, Nrf2 was activated by Cd in SCs as demonstrated by the increased expression and nuclear translocation of this transcriptional protein (Figure 3A,B). Its activity controls groups of genes, more than 250 in total, involved in the antioxidant defense and detoxification of electrophiles, as well as in intermediary metabolism, lipid biosynthesis and pentose phosphate pathway regulation. The entire series of genes associated with the biosynthesis, redox restoration, and detoxification function of cellular glutathione presents a consensus sequence in their promoter for this transcription factor.^37^ In this respect, our results demonstrated that the expression of a series of proteins decoded by these genes is upregulated in SCs by the treatment with Cd, including xCT, GCLC, and GSTP (Figure 3), ultimately leading to a dose‐dependent induction of Cys uptake and utilization in the biosynthesis and redox function of GSH (Figure 4). These changes in the metabolism of cellular glutathione are the signature of the adaption stress response elicited by the prooxidant effect of Cd in SCs. Also, our study demonstrates that this stimulation of GSH metabolism resulted in an increased efflux of this tripeptide and other thiols in the extracellular space. The same type of response is observed in other cell models exposed to different types of electrophiles and cellular stressors,^45^, ^52^, ^59^ thus representing a physiological response to oxidative stress to increase the availability of thiols to the extracellular protection systems and to other cells.^37^ However, a sustained activation of Nrf2 and GSH metabolism may lead to adverse cellular effects inducing allostatic overload and reductive stress, a dysmetabolic condition resulting in an exccess of reducing equivalents and altered function of redox‐sensitive nodes in cellular pathways.^60^

Accordingly, at the cellular level, the Cd‐induced stimulation of GSH metabolism was associated with a marked repression of protein S‐glutathionylation (Figure 3I). Such posttranslational modification of cellular proteins is regulated by the activity of specific enzymes, including glutaredoxins, glyoxilases, and GSTs,^37^, ^61^ important in both the transduction of cellular signals and protection of Cys residues against overoxidation and damage.^45^, ^61^ The PSSG enhancing activity of MLT was not effective to restore the levels of this protein posttranslational modification of porcine SCs treated with Cd (Figure 3F). Also, MLT did not restore the upregulation of GSH biosynthesis and efflux, and the increased expression of proteins that support these processes, such as the cystine transporter xCT and the GSH biosynthesis enzyme subunit GCLC. These may thus represent long‐lasting responses to Cd toxicity that do not directly intervene in the observed cytoprotective effect of MLT in porcine pre‐pubertal SCs. Conversely, MLT significantly corrected the Cd‐induced activation of Nrf2 activation and the induction of GSTP protein expression, suggesting a specific role for these proteins in the cytoprotective response to the reductive stress of Cd in SCs. GSTP is a Nrf2‐dependent gene, but other transcription factors are involved in the redox‐dependent modulation of its expression (reviewed in^34^, ^52^), such as NFkB and AP1, an heterodimeric protein composed of proteins of different families, including cJun.^62^ GSTP is one of the main isoforms of a family of transferases important in the phase II response to xenobiotics and in general in the detoxification of cellular electrophiles by their S‐conjugation with cellular GSH.^63^, ^64^ Also, GSTP is reported to possess thiol isomerase activity and to catalyze the glutathionylation of accessible Cys residues of cellular proteins,^61^, ^65^ a function that does not appear to intervene in the cytoprotective effect of MLT of SCs exposed to Cd (discussed earlier). These results suggest that the reduction of other functions of GSTP, but not its S‐glutathionylation activity, may have a role in the cytoprotective effects of MLT in pre‐pubertal porcine SCs exposed to Cd toxicity. In this respect it is important to consider that GSTP is a redox chaperone forming a regulatory interactome with several proteins important in the adaptive stress response, inflammation and death programs activation. These proteins include the transcription factors and cellular kinases modulated by MLT in this study on Cd‐treated SCs, including Nrf2, NFkB, AP‐1, and Akt, respectively (reviewed in Bartolini et al.^52^).

NFkB activity appears to be central to MLT signaling and cytoprotective function, occurring by gene modulation of main cellular antioxidants, including CAT and GSH. NFKB is involved in the testicular protection effects of MLT demonstrated in an animal model of LPS‐induced inflammation and oxidative stress,^51^ and together with cellular kinases important in MLT signaling, such as Akt and the survival MAPK ERK1/2,^19^, ^20^ the activity of this transcription factor is modulated in liver cells during MLT protection against H_2_O_2_‐induced oxidative stress.^36^

In the present study, MLT, at the same time synergized with Cd to induce NFkB and Akt signaling, and antagonized Cd in activating Nrf2. Available data do not allow to establish a cause‐effect relationship between these molecular responses to MLT, but it is conceivable to speculate that both of them may play a role in alleviating the reductive stress of SCs during Cd treatment. Furthermore, depending on the intensity of the stress response by the increasing concentrations of Cd (namely 5 and 10 μM), different types of genes can be involved in the cytoprotective effect of MLT. The H_2_O_2_‐metabolizing enzyme CAT was involved at the lower Cd concentration (Figure 1), whereas GSH‐related genes were involved at the higher one (Figure 4). These differences in the gene response to MLT, can be explained by means of the exponential growth in H_2_O_2_ production and efflux when the dose of Cd applied to SCs increased from 5 to 10 μM (Figure 1). Apparently, increasing the flux of H_2_O_2_ in SCs, different combinations in the activation state of NFkB and other transcriptional and signal transduction proteins are produced in these cells. These may include the component of AP‐1 transcriptional complex cJun,^62^ the expression of which was inhibited by MLT at 5 μM Cd and activated at 10 μM Cd (Figure 3D). Also, MAPK‐ERK1/2 signal transduction function may play a role in this respect. In fact, this kinase with key role in MLT signaling and cytoprotective function,^20^ was activated only at the lower dose of Cd and in correspondence to CAT activity induction. The role of these variable interactions between these multiple elements of MLT signaling thus demonstrate the functional pleiotropy of this cytoprotective agent^20^ and its potency in modulating the cellular metabolism of H_2_O_2_. In this respect, it cannot be ruled out that the gene response to MLT in SCs exposed to Cd toxicity, could directly affect the ROS‐generating systems of the cell. This may occur, for example, decreasing the levels of superoxide, that is the cellular precursor of H_2_O_2_, by a reduced mitochondrial leakage of electrons or membrane NADPH‐oxidase activity. At the same time, other ROS‐scavenging genes other than CAT may have a role in the antioxidant response to MLT of SCs, such as superoxide dismutase, glutathione peroxidases, and peroxyredoxins that are worth investigating further.

In conclusion, Nrf2 transcription factor activation and GSTP protein upregulation are molecular events associated with the reductive stress response to Cd toxicity in porcine pre‐pubertal SCs. MEL was demonstrated to revert these effects of Cd toxicity affecting antioxidant and detoxification genes associated with the modulation of redox‐sensitive transcription factors and cellular kinases, such as NFkB, cJun, MAPK‐ERK1/2, and Akt, respectively. These aspects described for the first time in these cells, warrant further preclinical and clinical investigation holding potential in the treatment of Cd‐induced testicular damage and fertility problems.

## AUTHOR CONTRIBUTIONS

Desirée Bartolini, Iva Arato, Francesca Mancuso, Francesco Galli, and Giovanni Luca conceptualized and designed the study. Desirée Bartolini, Iva Arato, Francesca Mancuso, and Daniela Giustarini performed most of the experiments, methodology and the acquisition of the data, drafted the initial manuscript, and approved the final manuscript as submitted. Carmine Vacca, Catia Bellucci, Maria Chiara Aglietti, and Anna Maria Stabile provided technical help. Gabriele Cruciani, Giovanni Luca, Ranieri Rossi, and Francesco Galli contributed reagents and acquisition of funding. Mario Rende, Gabriele Cruciani, Francesco Galli, Riccardo Calafiore, Ranieri Rossi, and Giovanni Luca critically reviewed and revised the manuscript. All authors approved the final manuscript as submitted.

## CONFLICTS OF INTEREST

The authors declare no conflicts of interest.

## Data Availability

The data that support the findings of this study are available from the corresponding author upon reasonable request.
